# Adaptations to the One-Child Policy: Chinese Young Adults’ Attitudes Toward Elder Care and Living Arrangement After Marriage

**DOI:** 10.3389/fpsyg.2021.608111

**Published:** 2021-03-03

**Authors:** Xiaochen Chen, Cuo Zhuoga, Ziqian Deng

**Affiliations:** Department of Psychology, Renmin University of China, Beijing, China

**Keywords:** elder care, Chinese only children, attitudes toward nursing homes, living arrangement, young adults

## Abstract

After four decades of China’s family planning policy, the shrinking family size and increasing life expectancy pose special challenges for the one-child generation in terms of providing care for aging parents. The current study explored young adults’ responses to such pressure by examining their concerns about elder care, attitudes toward nursing homes, and living arrangement after marriage in a sample of 473 Chinese working young adults from six cities in China (46.9% males, *M_age_* = 25.1 years, 47.8% only children). Results showed that although most of the young adults reported to have thought about the issue of parents’ elder care, the majority did not worry a lot about it. Only children expressed similar levels of worrying as those with siblings did. However, educational level, rather than sibling status, was systematically related to concerns about parents’ elder care and attitudes toward nursing homes. People with higher education tended to worry less about elder care, and were less likely to consider placing parents in nursing homes as a violation of filial piety. Analyses of the married sub-sample (*n* = 140) revealed that only children were more likely to co-reside with parents after marriage than those with siblings. And the main reason for co-residence was that the younger generation needed their parents’ help for childcare, rather than to better take care of their parents. Implications for parents’ elder care among Chinese only children were discussed.

## Introduction

Family support for the elderly has long been the primary mode of elderly support in Chinese society. However, after four decades of China’s family planning policy, the shrinking family size and increasing life expectancies pose special challenges for the only-child generation in terms of providing elder care for their parents (Feng et al., 2014). Despite widespread concerns about only children’s readiness for shouldering their filial duty of parental old-age care (e.g., Feng, 2015), empirical studies on how singleton status could affect young people’s concerns, values, and plans for parental elder care are scarce. For example, compared to people with siblings, do only children worry more about taking care of their parents? Do they hold more positive attitudes toward institutional care due to lack of inner-family help? Are they more likely to live together with parents after marriage to better take care of their parents? The current study attempted to provide preliminary answers to these questions by comparing only children and those with siblings in a sample of Chinese working young adults.

### Parents’ Elder Care: Challenges for Only Children

For centuries, filial piety (*xiao*) had been the primary virtue in Chinese families (Deutsch, 2006). According to the values of filial piety, children should respect their parents, obey them, and are obligated to care for elderly parents and respond to their needs. Traditional Chinese family roles were also highly gendered, and families were patrilineal (Zhan and Montgomery, 2003). Sons were expected to provide for their aging parents and carry on the family name, whereas daughters were expected to leave their natal families after marriage, and their family obligations were transferred to their husbands’ parents. Despite dramatic socio-economic changes taking place in China, recent research has shown that the core value of filial piety is still highly valued by young people (e.g., Gui and Koropeckyj-Cox, 2016; Liu and Li, 2020).

However, the one-child policy (OCP) has posed new challenges to family-based elderly support. For generations before the implementation of family planning policies, the overwhelming majority of Chinese couples had more than one child. Therefore, when parents needed care, siblings could share both financial and caregiving tasks while organizing the care of aging parents (Zhan, 2002). In contrast, the only-child generation faces the obligation of parent care without siblings. In addition, rapid economic change and intensifying competition in the workplace have placed more pressure on today’s Chinese young adults to succeed in education and work compared with previous generations (Gui and Koropeckyj-Cox, 2016). Taken together, the lack of inner-family help and increased conflicts between work and family roles (Cheung et al., 2006) create significant challenges for the first generation only children to fulfill their filial obligations to provide family-based elder care for their parents.

How are adult only children going to cope with the practical issue of parent care has called widespread concerns. On the one hand, despite evidence contradicting the “little emperor” stereotype of Chinese only children (e.g., Falbo and Poston, 1993; Xiao and Feng, 2010), there is still lingering doubt about this generation’s capabilities and a feeling that the OCP has created a spoiled generation of singletons with personality and behavioral deficiencies (Cameron et al., 2013), and that when this generation grows up, they may be less dutiful in providing parents’ old-age support than people with siblings. On the other hand, emerging evidence from qualitative research has revealed that only children, who have grown up in an environment characterized by high parental expectations and heavy investment, seemed to feel especially responsible for their parents’ old age support because of their singleton status (Deutsch, 2006; Liu, 2008; Gui and Koropeckyj-Cox, 2016). More empirical studies are thus needed to examine how singleton status would affect these young people’s concerns as well as their personal plans (e.g., use of institutional care) for dealing with the impending issue of parents’ elder care.

### The Role of Gender and Education

The OCP may also challenge traditional patrilineality in China. If parents have only one child and that child is a daughter, the parents may have to depend on her for affective ties and their future economic welfare. There is evidence documenting equal treatment of sons and daughters among parents of only children (Fong, 2002; Deutsch, 2006). Although historically sons have been primarily obliged to care for aging parents, in the absence of sons, daughters’ attitudes and behaviors regarding elder care may change. It is reasonable to expect that if any gender differences in attitudes toward elder care were found among young adults, the differences would be reduced or even disappear among only children.

A similar rationale applies to young people’s living patterns after marriage. Existing evidence shows that the traditional pattern of living with the husband’s family has changed. Although the majority of young married couples in urban areas now live alone (Feng, 2006; Wang, 2013), a survey with married only children revealed that those who did not live alone tended just as much to live with the parents of the wife as with the parents of the husband (Feng, 2006). However, to what extent young people’s choice of co-residence with parents after marriage relates to issues of elder care remains to be examined.

In addition, one unintended consequence of the OCP has been the increase in children’s education investment (Fong, 2002; Wang et al., 2020). More and more Chinese children, especially those who are the only child in the family, are going to college or even graduate school. However, little is known about whether this increased education level will positively impact future elder care. Existing studies have shown a mixed picture. On the one hand, a study with middle-aged participants from six Chinese cities showed that education counteracted the impact of modernization and served to sustain the traditional value of filial piety (Cheung and Kwan, 2009). On the other hand, Zhan (2004) found that higher educational levels were negatively related to young people’s commitment to parental care when they foresaw job and care conflicts, since education provides a person with greater geographic mobility and higher achievement orientation sometimes in defiance of their filial obligations (Deutsch, 2006). The intricate role of education on the one-child generation’s attitudes toward filial obligations and their plans for elder care warrants further investigations.

### The Current Study

The current study attempted to address the above-mentioned gaps by examining Chinese young adults’ attitudes toward issues of parents’ elder care, with an emphasis on comparing only children and those with siblings. More specifically, we examined young adults’ concerns about elder care, attitudes toward nursing homes, and living arrangement after marriage. Findings from the current study could shed light on future preparations of elder care as more and more only children reach the age of having to shoulder their filial responsibilities of parents’ old-age support.

## Materials And Methods

### Participants and Procedures

As part of a larger research project on youth development in China, data were collected in 2007 from young workers who were employed in six cities, collecting data from about 100 workers per city. The six cities were selected in terms of size (i.e., large and small) and section of China (i.e., eastern, western, and central). Specifically, the large cities were in the eastern part of China, Nanjing (Province: Jiangsu), in the west, Lanzhou (Province: Gansu), and in central China, Changchun (Province: Jilin). The small cities were in the east, Jinhua (Province: Zhejiang), in the west, Anshun (Province: Guizhou), and in central China, Ezhou (Province: Hubei). Within each city, companies (*danwei*) that offered jobs within the 15 most common urban occupational sectors were identified (Department of Population and Employment Statistics, 2005). These occupational sectors were, ranging from those yielding the largest to the smallest number of jobs: manufacturing, hotel and catering services, public services, transportation and communications, wholesale and retail trade, education, public administration, data and computer services, leasing and business services, construction, public health and social services, utilities (including electricity, gas, and water), banking, sports and entertainment, and real estate.

Once the companies were identified, trained graduate research assistants (RAs) approached the administrators of these companies and sought permission to administer a paper and pencil questionnaire to some of their younger workers. The RAs relayed that the purpose of the study was to “find out what life and work is like for young adults.” Before completing the questionnaire, participants were informed that they would not be asked to identify themselves or their company on the questionnaire. If the company employed many young adults, the RAs administered the questionnaire to only a randomly selected subset of their younger workers. In some instances, when the company was small, the RAs administered the questionnaire to all the company’s young-adult workers. The participants completed the questionnaire at their workplace while an RA waited, answering questions as needed. Completing the questionnaire took about 15 min and each participant was given a token gift (valued at about US $1) for completing the questionnaire.

As part of the research protocol, participants were asked about their sibling status and the type of locality of their hometown (i.e., city or countryside). Since most of the only children came from cities (*n* = 226, 91%), and to better disentangle sibling status from geographical origin, we limited our analyses to the urban subsample. As shown in Table 1, the final analytic sample consisted of 473 participants (53.1% female), ranging in age from 17 to 31 years (*M_age_* = 25.1 years, *SD* = 3.3). About 15% of the participants had been born before the OCP (i.e., born before 1979). Forty-eight percent of the participants had grown up without siblings, and 69.6% had never been married. The educational attainment of the sample ranged from middle school completion to having graduate degrees, with 5.9% indicating middle school completion, 21.8% high school completion, 27.3% junior college, 42.1% having a bachelor’s degree, and 3.0% having a graduate degree.

**Table 1:** Characteristics of the sample (*n* = 473).

Variables	*n*	percent
Age (*M* = 25.1, *SD* = 3.3)
Born before the OCP	74	15.6
Born in or after the OCP	399	84.4
Gender
Male	222	46.9
Female	251	53.1
Sibling status
Only child	226	47.8
Sibling(s)	247	52.2
Marital status
Unmarried	329	69.6
Married	140	29.6
Divorced	3	0.6
Education level
Middle school	28	5.9
High school	103	21.8
Junior college	129	27.3
4-year college	199	42.1
Graduate school	14	3.0

Percentages may not sum up to 100% due to missing data.

### Measures

#### Concerns for Elder Care

Young adults’ concerns regarding parents’ elder care were assessed by three items. Participants were first asked whether they had ever thought about the issue of their parents’ old-age support, if they answered “yes,” they were further asked to rate the degree to which they worried about it on a 4-point Likert scale (1 = *not at all* and 4 = *worry a lot*). The third item assessed young adults’ thoughts about challenges to elder care. Participants were presented with a list of difficulties that their parents might encounter in their later lives (i.e., care when sick, care for daily routine, lack of financial aid, lack of spiritual support, lack of interpersonal communication, and others). They were asked to select the one that they believed would be most difficult to deal with.

#### Attitudes Toward Nursing Homes

Two aspects of attitudes toward institutional care were assessed.

##### General Attitudes

General attitudes toward using nursing homes for parents’ elder care were assessed using four items. The first two items focused on the conflict between institutional care and the traditional value of filial piety (e.g., “Sending parents to a nursing home is a way to shrug off filial responsibilities”), while the last two items captured the practical value of institutional care as a means for elder care (e.g., “It is probably the most practical way to solve the problem of taking care of the elderly”). Participants were asked to rate whether they agreed with each of the four statements (0 = *disagree* and 1 = *agree*). To simplify the analysis, scores on the first two items (*α* = 0.84) were summed up to create an indicator of *violation of filial piety*. Similarly, scores on the last two items (*α* = 0.67) were summed up to create an indicator of *practical solution*.

##### Personal Plan

The participants’ personal plan regarding sending parents to a nursing home was assessed using the following item: “Your parents may need others to take care of them when they get old. However, you have your work and your own family. In this case, would you think about sending your parents to a caring house?” Responses were given on a 5-point Likert scale (1 = *definitely would not think* and 5 = *definitely would think*).

#### Living Arrangement After Marriage

Married young adults were asked to identity their current living arrangement from the following choices: living in a separate apartment, living together with the husband’s parents, living together with the wife’s parents, and other. For those living together with parents, reasons for co-residence were further explored: participants were presented with a list of possible reasons (e.g., “We need parents’ help for child care” or “We cannot afford an apartment, so we have to live together with parents”), and were asked to check reason(s) (up to two reasons) that applied to them.

#### Relations With Parents

Young adults’ relations with parents were treated as a control variable in the current study since previous research with the elderly showed that emotional closeness between parents and children could affect the preferred way of elderly care (Tao and Liu, 2019). Participants were asked to rate how they were getting along with their fathers and mothers separately (1 = *poorly* and 5 = *pretty well*). Scores on these two items were averaged to get the indicator of relations with parents (*α* = 0.63).

## Results

### Analytic Plan

The analyses were divided into three parts. First, descriptive analyses were carried out to explore young adults’ concerns regarding parents’ elder care. Next, multiple regression analyses were conducted to examine how singleton status, along with other demographic variables, could affect young people’s concerns over (i.e., degree of worry) parents’ old-age support as well as their attitudes toward institutional care. In this set of regression analyses, singleton status (people with siblings as the reference group) was first entered into the model. Then, age, gender (female as reference), educational level, marital status (unmarried as reference), and relations with parents were entered to examine whether additional covariates change the singleton status effect. In the third step of the regression analyses, gender by singleton status interactions were entered to explore possible gender differences in sibling effects on attitudes toward elder care. The final set of analyses was more exploratory in nature. With the trimmed sample of married young adults (*n* = 140), we examined whether singleton status could affect living arrangement after marriage and how that would relate to parents’ elder care.

### Concerns for Parents’ Elder Care

**Figure 1:**
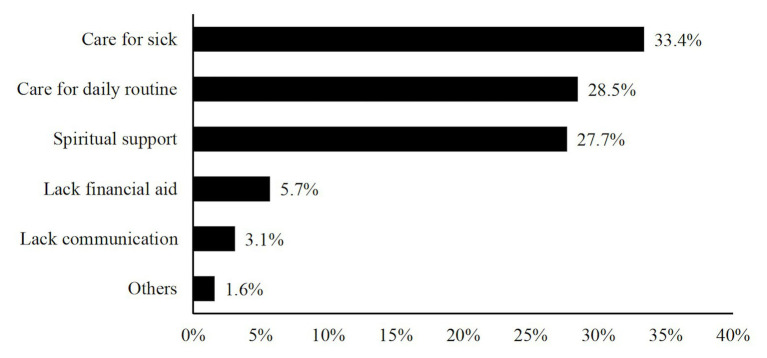
Most challenging aspect in parents’ later lives.

Over 80% (82.4%) of the participants reported having thought about the issue of parents’ elder care. Only children were as likely to think about this issue as people with siblings, *χ*^2^(1) = 0.13, *ns*. As for major difficulties in parents’ later lives (shown in Figure 1), “care for sick” was the most frequently selected (rated by 33.4% of the participants), followed by “care for daily routine” and “lack of spiritual support” (rated by 28.5 and 27.7% of the participants, respectively). “Lack of financial aid” and “lack of interpersonal communication” were least chosen as major challenges. The distributions of expected difficulties in parents’ later lives were similar among only children and people with siblings, *χ*^2^(5) = 4.13, *ns*.

Although most participants reported having thought of parents’ elder care, the majority of them did not worry a lot about it. Specifically, 15.0% of them reported “not worried at all,” about half (46.4%) “not so worried,” 31.1% “worried,” and only 7.4% “worried a lot.” To systematically examine how singleton status could affect the participants’ concerns regarding parents’ elder care, multiple regression analyses were carried out, with degree of worrying as a continuous outcome variable. As shown in the left column of Table 3, neither singleton status nor the interaction terms between singleton status by gender were significant. Notably, educational level was found to be a significant predictor of the *degree of worry* (*b* = −0.08, *p* < 0.05). People with higher educational attainment tended to worry less about their parents’ elder care.

### Attitudes Toward Nursing Homes

As shown in Table 2, about two-thirds of the participants did not agree that sending parents to a nursing home was against the value of filial piety, nor did they think the use of institutional care was a way to shrug off filial responsibilities. About half of the participants agreed with the statements describing institutional care as a practical solution for parents’ elder care. No significant results from Chi-square tests (all *ps* > 0.05) suggested similar response patterns among only children and people with siblings.

**Table 2:** Descriptive statistics on attitudes toward nursing homes (%).

Sending parents to a nursing home is…	All young adults	Only child	People with siblings	*χ*^2^(*df*)
Against filial piety				0.00 (1)
Agree	30.7	30.7	30.8	
Disagree	69.3	69.3	69.2	
Shrug off filial responsibilities				1.16 (1)
Agree	37.7	40.2	35.4	
Disagree	62.3	59.8	64.6	
Fact have to face in the future				0.69 (1)
Agree	52.2	54.2	50.4	
Disagree	47.8	45.8	49.6	
Most practical solution				1.05 (1)
Agree	45.7	48.2	43.5	
Disagree	54.3	51.8	56.5	
Probability of use nursing homes				2.68 (4)
Definitely would not	51.1	48.9	53.0	
Probably would not	19.5	19.6	19.4	
Not sure	14.6	15.6	13.8	
Probably would	13.3	15.1	11.7	
Definitely would	1.5	0.9	2.0	

Next, regression analyses were carried out to further examine the factors influencing attitudes toward nursing homes. As mentioned in Materials and Methods, scores on the two items about violations of filial piety were summed up and treated as a continuous variable (ranging from 0 to 2). Similar procedures were also carried out to get a continuous indicator of nursing homes as a practical solution to elder care. As shown in the middle columns Table 3, there were significant gender differences in attitudes toward nursing homes. Compared with females, males were more likely to view sending parents to a nursing home as a violation of filial piety (*b* = 0.17, *p* < 0.05), and were less likely to consider institutional care as a practical solution to parents’ elder care (*b* = −0.18, *p* < 0.05). Educational level was also a significant predictor of attitudes toward nursing homes. People with higher educational attainment were less likely to view sending parents to a nursing home as a violation of filial piety (*b* = −0.11, *p* < 0.05) and were more likely to agree with the idea that the use of nursing homes was a *practical solution* for taking care of aging parents (*b* = 0.11, *p* < 0.05). In addition, participants who had better relations with their parents were less likely to consider nursing homes as a practical solution for elder care (*b* = −0.23, *p* < 0.01). Contrary to our expectations, singleton status was a not a significant predictor in the models predicting attitudes toward institutional care, nor were the interaction terms between singleton status by gender.

**Table 3:** Regression analyses predicting degree of worrying and attitudes toward nursing homes.

	Degree of worrying		General attitudes toward nursing homes				Personal plan	
			Violation of filial piety		Practical solutions		Use of nursing homes	
Predictor	*B*	*SE*	*B*	*SE*	*B*	*SE*	*B*	*SE*
Step 1
Only child	0.12	0.11	0.04	0.12	0.10	0.11	0.09	0.15
Step 2
Age	−0.02	0.02	−0.02	0.02	0.02	0.02	0.03	0.02
Gender	−0.10	0.11	0.17^*^	0.08	−0.18^*^	0.09	−0.20	0.15
Education	−0.08^*^	0.04	−0.11^*^	0.05	0.11^*^	0.04	−0.06	0.06
Married	0.10	0.10	−0.14	0.11	0.15	0.11	−0.01	0.14
Divorced	−0.62	0.48	0.47	0.52	−0.80	0.50	−1.06	0.68
Relwtparents	−0.11	0.06	0.11	0.07	−0.23^**^	0.07	−0.17	0.09
Step 3
Only child × gender	0.06	0.15	0.08	0.16	−0.06	0.16	0.12	0.21

Relwparents = relations with parents.

* *p* < 0.05;

** *p* < 0.01.

Paradoxically, despite group differences in general attitudes toward institutional care, for personal plans of using a nursing home for parents’ elder care, none of the predictors in the model was significant (see right column in Table 3). A closer examination of the data revealed that more than half (51.1%) of the participants reported that they “definitely would not” send their parents to a nursing home, while another 19.5% reported that they “probably would not” do so. On the other hand, 14.6% were “not sure,” and only a few answered that they “probably would” (13.3%) or “definitely would” (1.5%) send their parents to a nursing home.

### Living Arrangement of the Married Sub-Sample

This exploratory study further examined the current living arrangement and corresponding reasons of married young adults (*n* = 140). About two-thirds (63.6%) of the participants lived separately from parents after marriage; 29.3% of married participants reported living together with the husbands’ parents, whereas 4.3% of married young adults lived together with the wife’s parents. Patterns of living arrangement differed across singleton status, *χ*^2^ (3) = 8.20, *p* < 0.05. As shown in Table 4, although the most common living arrangement for both only children and people with siblings was living in a separate apartment, a larger proportion of singletons reported co-residence with parents. Noticeably, it was among only children that a few participants reported living together with the wife’s parents after marriage, which is contrary to Chinese traditions.

**Table 4:** Living arrangement after marriage by singleton status (%).

Living arrangement	Singleton status		
	Only child (*n* = 62)	People with siblings (*n* = 78)	Total
Separate residence	60.0	66.2	63.6
With husband’s parents	26.7	31.2	29.3
With wife’s parents	10.0	0.0	4.3
Other	3.3	2.6	2.9

**Figure 2:**
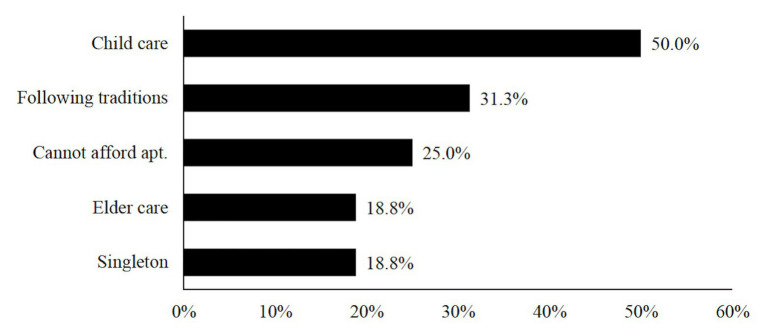
Reasons for co-residence with parents after marriage.

As to reasons for living together with parents (see Figure 2), the most frequently rated one was “need parents’ help for childcare” (50.0% of the respondents), followed by “following the Chinese tradition to live with the husband’s family” (31.3% of the respondents). In addition, one-fourth (25.0%) of married young adults mentioned “cannot afford our own apartment.” Contrary to our expectations, “to take care of parents” and “one side of the married couple is an only-child” were least chosen as reasons for living together with parents (each was chosen by 18.8% of the respondents).

## Discussion

As the first generation of only children enters adulthood and starts to establish their own families, issues of elder care of this generation’s parents have attracted much attention among social science researchers. However, although existing studies by demographers and sociologists have primarily focused on intentions for old-age support from the perspective of parents (e.g., Gustafson and Huang, 2014; Shan, 2016; Tao and Liu, 2019), less is known about first-generation only children’s concerns, values, and future plans regarding their parents’ elder care. The current study attempted to fill this gap by surveying a large sample of working young adults from six Chinese cities. We emphasize two lines of findings that make original contributions to the literature.

### Singleton Status and Attitudes Toward Elder Care

Are only children really “spoiled brats” wanting to shirk future responsibilities of parental care? We believe that the answer, based on this study’s findings, is no. As a matter of fact, only children expressed similar levels of concerns about elder care as did people with siblings. This finding may be interpreted in two ways. On the one hand, a child-centered parenting approach may foster closer emotional ties between only children and their parents (Deutsch, 2006; Feng et al., 2014), which in turn may lead to a stronger sense of filial responsibilities among singletons to take care of aging parents. On the other hand, being the only ones available to care for parents in the future, only children may feel obligated to shoulder the responsibilities because they have no other choice.

Despite the emerging sense of the burden of parents’ old-age support, most of our participants, irrespective of whether they were singletons, did not seem to worry a lot about this issue for now. Contrary to our expectations, only children were not more open to institutional care than were people with siblings. Consistent with the findings of a previous study (Song and Huang, 2011), we found that although only children were more likely to live together with parents after marriage, the main reason for co-residence was to obtain parents’ help for childcare, rather than to better take care of aging parents. These findings suggest that although only children would like to accept filial responsibilities of elder care, they are not yet fully prepared with practical solutions to fulfill this duty. It should be noted that most of our young-adult participants had never been confronted with actual problems of reconciling their parents’ elder care needs with their own family and professional lives as most of their parents were still in their late middle age and in good health. Future research is needed to examine how the only-child generation responds to the challenges of elder care as their parents grow older.

### Ambivalent Attitudes Toward Institutional Care

Due to reduced family size and increased job-care conflicts, the only-child generation may need to seek help outside their families, including formal caregiving institutions. However, sending parents to a nursing home contradicts the traditional value of *xiao*, which requires family-based elder care. Therefore, it is of particular importance to examine possible attitudinal changes or adaptations toward institutional care in relation to the long cultural practice of *xiao*. The current findings demonstrated ambivalent attitudes toward institutional care among Chinese young adults despite their singleton status. There was a discrepancy between the participants’ general attitudes and personal plans regarding institutional care: While many did not agree that sending elderly parents to a nursing home was against filial piety, only a small proportion of them would like to turn to institutional care for their parents in the future. This pattern of findings indicates a shift in beliefs about fulfilling filial responsibilities. However, the value of *xiao* is so entrenched in Chinese society that adult children who seek out nursing home care for their aging parents would probably encounter considerable moral pressure from others against doing so (Chang and Schneider, 2010; Feng and Jiang, 2014), which would make them feel reluctant or hesitant to use institutional care.

Regression analyses on attitudes toward nursing homes yielded significant gender differences. And the non-significant interaction between gender and singleton status suggested similar gendered patterns among only children and people with siblings. In general, males held more negative attitudes toward institutional care. Despite recent literature documenting increased involvement in parental care among daughters (see review by Fu et al., 2016), current findings suggest that the gendered expectations about filial responsibilities may still have lingering effects on the one-child generation. Traditionally, sons had been assumed to be primarily responsible for family-based old-age support. Perhaps, deep-rooted culture values render institutional care less acceptable among males no matter they have siblings or not.

In addition, educational attainment was shown to be linked with young people’s concerns about elder care and attitudes toward nursing homes. People with higher educational levels expressed less worries about parents’ old-age support and were more open to institutional care. It is plausible that higher education could lead to more information about and better financial preparations for care alternatives, which helps to relieve the sense of burden of elder care. Existing evidence has shown that the high cost of professional care in institutions contributes to a shift in attitudes about institutional elder care from stigma to privilege (Zhan et al., 2008).

### Limitations and Future Directions

The findings of the present study are inconclusive without further research and more data. First, only children in the current sample were from urban areas. Since China has separate support tracks for urban and rural residents (i.e., providing urban residents with better pensions and medical care security), one would expect that only children who grew up in the countryside may feel particularly pressured to shoulder parental care responsibilities. A sample with a proportional urban and rural balance is desirable for future studies. Second, as a preliminary attempt to examine first-generation only children’s attitudes toward elder care, the models presented in the current study were relatively simple. We concede that for each family, decisions on elder care arrangements are complicated processes, which may depend on a myriad of factors concerning the characteristics of multiple generations of family members. Larger samples with a fuller array of demographic variables (e.g., parents’ health condition, income, and marital status) in future studies could help provide a more nuanced understanding of how singleton status affects people’s attitudes and behaviors concerning elder care. Moreover, as an exploratory study, measures employed in the current study were simple. More sophisticated scales were needed in future research to better capture relevant psychological constructs, such as values on elder care. A related issue is that the current study is descriptive in nature. Future research would benefit from more process-oriented measures, such as measures capturing parent-child affective ties, or children’s self-construes, which may help explain mechanisms underlying the linkage between singleton status and attitudes toward parents’ elder care.

Last but not least, since participating young adults were not yet fully engaged in their role of old-age support for their parents, the current study mainly focused on their concerns, values, and plans rather than their actual practices concerning elder care. Future research is needed to investigate this process when their transition to adulthood is complete and when their parents experience actual need of care.

### Practical Implications

Despite the aforementioned limitations, we believe that the current study has significant practical implications. The current findings suggest that first-generation only children have sensed the burden of elder care they will probably encounter in their middle age, but they are still ambivalent about how to deal with these challenges given the prevailing cultural norm of filial piety and structural constraints of reduced family size and increased personal pursuit. As Mills (1959) proposed, what people often perceive as personal troubles are in fact indicative of larger social issues. It is not realistic to expect young people from the one-child generation to solve their family problems by themselves, nor is it fair to expect individuals to solely shoulder the consequences of national population policies and demographic changes. It is imperative for public policy makers to improve the availability of various elder care services, such as hired home care, adult day care, and other community elder care service as well as the institutional care discussed. Ideally, such services could follow a family-like model and take care of older people just as their offspring would (Lan, 2002). Moreover, at the micro level, it would help if both adult children from the only-child generation and their parents be released from the cultural burdens to embrace alternative elder care resources.

## Data Availability Statement

The datasets generated and analyzed during the current study are available from the corresponding author on reasonable request.

## Ethics Statement

The studies involving human participants were reviewed and approved by the Ethics Committee of the Department of Psychology, Renmin University of China. The patients/participants provided their written informed consent to participate in this study.

## Author Contributions

All authors developed the conceptual background and designed the study. XC wrote the paper, while the other authors provided comments. CZ and ZD analyzed the data, and XC assisted with data analyses. All authors contributed to the article and approved the submitted version.

### Conflict of Interest

The authors declare that the research was conducted in the absence of any commercial or financial relationships that could be construed as a potential conflict of interest.
